# Use of Probiotics to Control Biofilm Formation in Food Industries

**DOI:** 10.3390/antibiotics12040754

**Published:** 2023-04-14

**Authors:** Andreia R. Tomé, Fábio M. Carvalho, Rita Teixeira-Santos, Mette Burmølle, Filipe J. M. Mergulhão, Luciana C. Gomes

**Affiliations:** 1 LEPABE—Laboratory for Process Engineering, Environment, Biotechnology and Energy, Faculty of Engineering, University of Porto, Rua Dr. Roberto Frias, 4200-465 Porto, Portugal; up201806129@edu.fe.up.pt (A.R.T.); up201502963@edu.fe.up.pt (F.M.C.); ritadtsantos@fe.up.pt (R.T.-S.); filipem@fe.up.pt (F.J.M.M.); 2 ALiCE—Associate Laboratory in Chemical Engineering, Faculty of Engineering, University of Porto, Rua Dr. Roberto Frias, 4200-465 Porto, Portugal; 3 Section of Microbiology, Department of Biology, University of Copenhagen, Universitetsparken 15, 2100 Copenhagen, Denmark; burmolle@bio.ku.dk

**Keywords:** biofilm, food industry, probiotic, anti-biofilm activity, displacement

## Abstract

Microorganisms tend to adhere to food contact surfaces and form biofilms, which serve as reservoirs for bacteria that can contaminate food. As part of a biofilm, bacteria are protected from the stressful conditions found during food processing and become tolerant to antimicrobials, including traditional chemical sanitisers and disinfectants. Several studies in the food industry have shown that probiotics can prevent attachment and the consequent biofilm formation by spoilage and pathogenic microorganisms. This review discusses the most recent and relevant studies on the effects of probiotics and their metabolites on pre-established biofilms in the food industry. It shows that the use of probiotics is a promising approach to disrupt biofilms formed by a large spectrum of foodborne microorganisms, with *Lactiplantibacillus* and *Lacticaseibacillus* being the most tested genera, both in the form of probiotic cells and as sources of cell-free supernatant. The standardisation of anti-biofilm assays for evaluating the potential of probiotics in biofilm control is of extreme importance, enabling more reliable, comparable, and predictable results, thus promoting significant advances in this field.

## 1. Introduction

According to Food and Agriculture Organization (FAO) and World Health Organization (WHO), probiotics are live microorganisms that provide health benefits to the host when ingested in adequate amounts [1,2,3]. *Lactobacillus* and *Bifidobacterium* are the most commonly used probiotic genera and are widely incorporated into functional foods (e.g., fermented dairy products) and dietary supplements [4]. Some health benefits associated with the consumption of probiotics include stimulation of the immune system, antagonism against gastrointestinal pathogens, cholesterol reduction, and anticancer effects [5]. Due to the highly documented beneficial effects of probiotics on human health, the food industry has become increasingly interested in these microorganisms.

Regulatory approaches used to approve probiotics in food vary across regions. In Europe, the European Food Safety Authority (EFSA) has developed the Qualified Presumption of Safety (QPS) approach, a safety assessment procedure for microbes found in food and feed chains. QPS includes microorganisms such as probiotics which have been assessed by the EFSA as not raising any safety concerns [6]. Moreover, Regulation (EC) No. 1924/2006 establishes directives on nutrition and health claims made on foods, covering the communication on the nutritional and health effects of probiotics [7]. In the US, the use of probiotics is regulated by the Generally Recognized as Safe (GRAS) guidelines developed by the Food and Drug Administration (FDA) [8].

**Figure 1:**
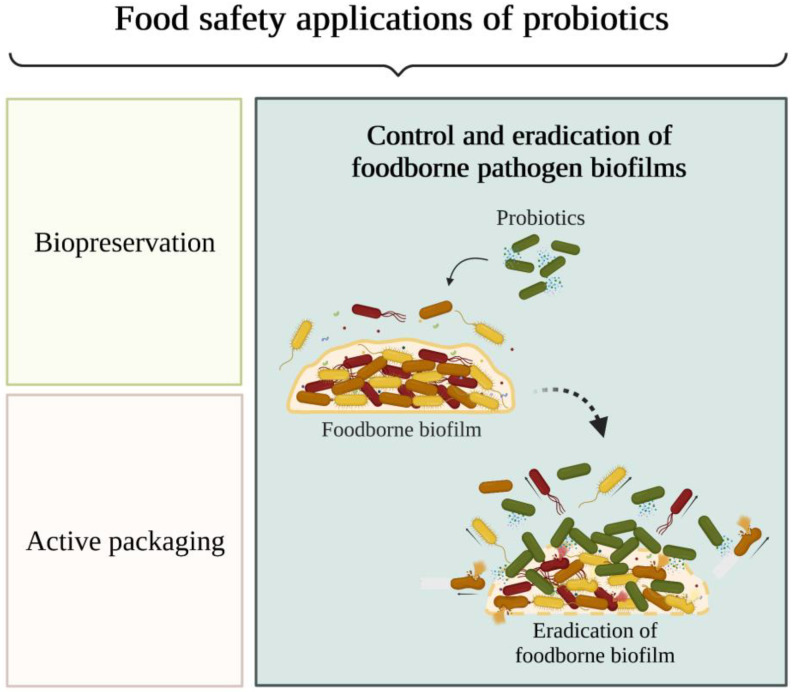
Food safety applications of probiotics: preservation, packaging, and control and eradication of foodborne pathogen biofilms.

In addition to their use in functional foods and dietary supplements, probiotics have been studied for applications in food safety assurance (Figure 1) as a promising strategy for food biopreservation, active food packaging, and prevention and control of biofilm formation in the food industry [5,9]. Biopreservation is a hurdle approach that is applied to extend the shelf life and prevent food spoilage using specific microorganisms and their antimicrobial products (e.g., organic acids, hydrogen peroxide, and bacteriocins) [9]. The combination of hurdles, such as probiotics and non-thermal technologies, can ensure that all pathogens are eliminated or rendered harmless in the final food product. Indeed, it has been reported that non-thermal techniques induce the formation of compounds that promote the growth and survival rates of beneficial microbes in food, making food products safer for consumption [10].

**Figure 2:**
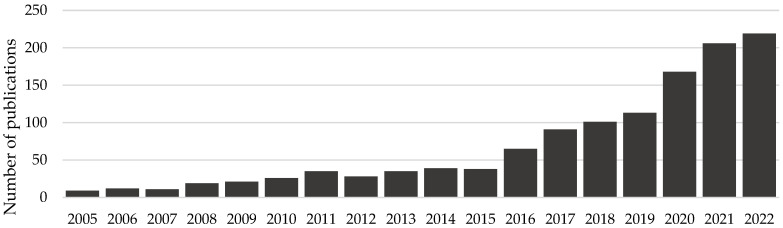
Number of scientific articles published on the PubMed database from 2005 to 2022 upon the search for “probiotic” and “biofilm” keywords.

The global probiotic market was worth more than US$ 48 billion in 2021, increasing 8% compared to 2020. In Europe, the market was € 9.4 million in 2021 [11]. Interestingly, the growth of the probiotic market has been accompanied by the growth of scientific papers on probiotics. For instance, in the PubMed online database, the search for the word “probiotic” returned 2800 publications from 2017 and practically twice as many publications (i.e., 5700) by 2022. In turn, the search for “probiotic” and “biofilm” returned only 9 publications from 2005, but 219 articles with these keywords were published by 2022 (Figure 2). 

This review aims to highlight the most recent and relevant studies on the performance of probiotics and their derivatives in the control of foodborne biofilms. To the best of our knowledge, this is the first work that focuses on the use of probiotics as a possible solution to displace pathogenic biofilms on food contact surfaces.

## 2. The Nature and Extent of Foodborne Diseases

When food safety is compromised, foodborne diseases can develop. They comprise a broad range of diseases caused by microorganisms, chemicals, toxins, radioactivity, or even physical agents present in ingested food or water. Foodborne illnesses constitute a public health problem since they boost morbidity and mortality, causing a significant number of hospitalisations and deaths [5,12,13]. Worldwide, the consumption of contaminated food or water results in 600 million infections (7.7% of the world’s population) and 420,000 deaths every year. In Europe, an estimated 23 million people suffer from foodborne diseases annually, resulting in approximately 5000 deaths [12]. The annual number of infections in the US is approximately 48 million, with more than 3000 deaths [14]. Additionally, foodborne diseases pose a huge economic burden. The lost productivity and medical costs associated with this type of infection are more than US$ 110 billion per year in low- and middle-income countries [15]. In the US, the total cost in 2018 was US$ 17.6 billion [16].

*Salmonella* spp. was the pathogenic microorganism causing the highest number of reported foodborne infections in 2020, followed by norovirus and *Listeria monocytogenes* [17]. Other important bacteria known to cause foodborne diseases include *Staphylococcus* spp., *Clostridium* spp., and *Vibrio* spp. [5]. For example, *Salmonella* can cause diarrhoea, fever, and abdominal pain; *L. monocytogenes* can cause febrile gastroenteritis and other extreme health problems such as meningitis and abortion; and *S. aureus* can cause acute gastroenteritis [18]. In 2020, campylobacteriosis and salmonellosis caused the highest number of reported zoonoses, followed by yersiniosis and intoxication by Shiga, a toxin produced by some *Escherichia coli* strains [17].

## 3. The Role of Biofilms in Food Contamination

Food contamination can occur at any stage of the food chain, from production, processing, storage, distribution, or even the preparation and cooking of food by consumers [19]. According to the European Council Regulation (EEC) No. 315/93, a contaminant is any substance not intentionally added to food which is present in such food as a result of the production, manufacture, processing, preparation, treatment, packing, packaging, transport or holding of such food, or as a result of environmental contamination [20]. Food contamination may lead to changes in taste, smell, texture, or appearance, which are considered unacceptable or undesirable [21]. Raw, uncooked, minimally processed food, mostly of animal origin, but also fruits and vegetables, are at high risk of bacterial contamination [22].

Instead of being present in the planktonic state, bacteria tend to adhere to food contact surfaces and form biofilms [23]. The risk of biofilm formation is particularly high in cutters, conveyor belts, drains, walls, and ceilings [24]. Biofilms pose a noticeable hygiene risk by being reservoirs of food pathogens and spoilage microorganisms, which alter the organoleptic properties of food by secreting lipases and proteases [23,25]. Additionally, biofilms have the ability to clog and corrode equipment, causing machinery technical failure and, consequently, huge economic losses [18,26]. The availability of nutrients from food residues and moisture on these surfaces promotes the development of biofilms, which can form in all types of materials, for instance, stainless steel, plastic, polystyrene, and glass [26]. The relevant biofilm-forming food-associated pathogens include *L. monocytogenes*, *Campylobacter* spp., *Salmonella* spp., *Pseudomonas* spp., and *E. coli* [22,23].

A biofilm can be defined as a complex aggregate of bacteria established in a three-dimensional structure and embedded in a matrix synthesised by the microbial community [24,27]. The generally accepted model of biofilm formation includes five stages: reversible attachment, irreversible attachment, maturation I, maturation II, and dispersion [28]. Initially, planktonic cells adhere to the surface via a single pole through weak and reversible interactions and can readily detach and return to the planktonic phase. Surface conditioning, which consists of organic substances absorbed on the surface, serves as a nutritional cue, thus triggering biofilm formation [26]. The transition from reversible to irreversible attachment occurs when bacterial appendages overcome physical repulsive forces, and consequently, cell surface proteins can permanently bind to the surface [26,29]. Cell attachment depends on multiple factors, including environmental conditions, surface characteristics, and microbial cell properties [23,30,31]. The maturation stage of biofilm formation encompasses the accumulation of cells, forming cell clusters that subsequently mature into microcolonies [28]. At this stage, bacteria increase the production of extracellular polymeric substances [26]. This extracellular matrix surrounds sessile microorganisms, protecting them from the stressful conditions found during food processing (e.g., low temperature, acidity, or oxidative and osmotic stresses) and limiting the effect of antimicrobials, which, in turn, allows biofilm growth and proliferation [13,31,32,33]. In addition, high cellular density facilitates direct horizontal gene transfer between cells, which can confer antibiotic resistance to bacteria within biofilms [24,34]. Cell dispersion can occur in mature biofilms; thus, bacteria can return to their planktonic form and colonise new surface locations [26,33]. Biofilm dispersion enhances microorganism spread, resulting in food contamination and spoilage [22]. The high cellular density and the concentration gradients of nutrients, oxygen, and waste cause biofilm cells to experience an ever-changing microenvironment. The original five-stage model of biofilm development presented before does not accurately represent these microenvironments or the complexity of biofilm structures and processes in real-world industrial systems. Thus, a recent publication proposed a more encompassing model for biofilm formation, which includes three steps: aggregation, growth, and disaggregation [35]. The suggested model considers different habitats and microenvironments, the possible influx of new cells, and surface-attached and non-surface-attached biofilms.

## 4. Biofilm Prevention and Control Strategies in the Food Industry

The best approach to eradicate biofilms in the food industry is to prevent their formation and, more importantly, prevent microorganisms from entering food processing facilities. The establishment of an effective hygiene protocol and a correct plant and equipment design are crucial to limit the access of microorganisms to factories and further contact with food [23,36]. For example, gaps, crevices, and dead areas should be avoided in order to minimise the locations where microorganisms can find shelter and grow [23]. The choice of surface materials and coatings is also important for inhibiting biofilm formation [36]. In addition, the adoption of a Hazard Analysis and Critical Control Point system (HACCP) is essential to preserve food safety and quality [24]. Its application by companies in the food sector is recommended in EC Regulation No. 852/2004 (Hygiene of Foodstuffs) and EC Regulation No. 853/2004 (Specific Rules Food of Animal Origin) of the European Union.

Once biofilms are formed on food contact surfaces, mechanical and physical cleaning actions are the first approaches to be applied [36], such as super-heated steam injection and high-pressure washing [26]. These measures disrupt the extracellular matrix, destroying the biofilm and moving the sessile cells to the sensitive planktonic state, which is essential for the complete removal of biofilms [33,36]. A combination of physical and chemical methods is commonly used in the food industry. For example, Cleaning-In-Place (CIP) combines mechanical actions with the use of chemical cleaning agents. Its efficacy depends on the properties of the surface being cleaned, the type of biofilm, the concentration of the cleaning agents, and CIP time and temperature [37].

Chemical treatment with detergents, sanitisers, and disinfectants seems to be an effective biofilm control method [23,31,38]. Sodium hypochlorite, hydrogen peroxide, peracetic acid, and sodium hydroxide are chemical agents that have demonstrated competence in reducing biofilms [22,25]. However, the doses and application times of these chemical agents are usually adapted to kill planktonic microorganisms, thus they may be inefficient against biofilms [24]. Additionally, biofilms are more tolerant to some biocides, including chlorine-based and quaternary ammonium sanitisers [25]. Several reasons have been suggested to explain this biofilm tolerance: (1) the defective diffusion of antimicrobials within the biofilm due to the extracellular polymeric matrix, (2) biocide enzymatic degradation within the matrix, (3) the slow metabolism of bacteria inside the biofilm, which prevents the effect of many antibiotics/biocides, and (4) the formation of spores, which have a high intrinsic resistance to environmental stresses. Additionally, the resistance of biofilms is enhanced because of acquired or intrinsic genetic modifications, including overexpression of efflux pumps and modification of antimicrobial binding sites [23,39].

Biofilm tolerance against synthetic antimicrobials and the negative consumer perception towards these chemicals, in addition to their release into the environment and the high amount of water spent in cleaning, have strengthened the search for different alternatives, namely for environmentally friendly disinfection [25,32]. Environment-friendly biofilm control strategies include the use of enzymes, bacteriophages, natural compounds such as essential oils, and bacterial products such as bacteriocins and biosurfactants [31,38]. Enzymes (e.g., proteases, lipases, and polysaccharidases) are biodegradable and low-toxicity bioactive macromolecules that have shown the ability to inhibit biofilm formation. Currently, they are widely used in detergents for application in the food industry, despite their production costs and strict time and temperature requirements [23,25,33]. Bacteriophages are viruses targeting prokaryotic cells and are, therefore, innocuous to humans. Some commercial solutions have bacteriophages in their composition due to their anti-biofilm ability, although they have some limitations in targeting microorganisms inside biofilms due to the extracellular matrix [25]. In turn, essential oils consist of a plant-derived mixture of secondary metabolites (e.g., phenol, thymol, and carvacrol) [33]. Some of their components exhibit important anti-biofilm properties, even though some essential oils may irritate the skin and other human organs [25]. Biosurfactants are amphipathic molecules secreted by microorganisms capable of disrupting the hydrophobic interactions involved in biofilm matrix cross-linking [33]. These metabolic products can also prevent biofilm formation by altering the hydrophobic characteristics of the bacterial surface and decreasing surface tension and, consequently, its adhesion ability [25]. Bacteriocins are ribosomally synthesised proteins or peptides with antibacterial activity [40,41]. For example, nisin, derived from *Lactococcus lactis*, has been approved for its antimicrobial activity against several foodborne microorganisms by the WHO and the FDA and is widely used as a food preservative [25,31]. The main disadvantages of these molecules are their high production cost and narrow-spectrum antibacterial activity [14].

Quorum sensing (QS) inhibition is believed to be another approach to control biofilm formation, although their relationship is not yet fully understood. Contrary to bactericidal strategies, molecules targeting QS cause less selection pressure to develop resistance to antimicrobial agents [22,25]. QS inhibition may occur through the following mechanisms: (1) competitive binding of inhibitors to QS signalling molecules (called autoinducers (AI)), (2) degradation of AI signals through quorum-quenching enzymes, (3) post-transcriptional control of QS genes via sRNAs, and (4) inhibition of AI. The disturbance of only one component of the QS pathway often leads to the downregulation of QS genes and the inactivation of the QS mechanism [25,42]. Quorum-quenching molecules are produced by microorganisms when they compete with neighbouring cells. However, bacteria can also degrade their own QS molecules to maintain an appropriate signal concentration [22].

Probiotics have arisen as a promising alternative strategy for controlling biofilm formation in the food industry, thereby preventing antimicrobial resistance associated with foodborne microorganisms [5].

### Probiotics as an Anti-Biofilm Approach

Several studies have shown that some probiotics, especially lactic acid bacteria (LAB), are able to prevent cell attachment and control biofilm formation by many pathogens [3,18,32]. This antagonistic activity may be due to competition for nutrients and adhesion sites or the release of antimicrobial metabolites such as bacteriocins, biosurfactants, organic acids, hydrogen peroxide, and inhibitory exopolysaccharides [18,24,30,32]. Furthermore, previous studies have shown the positive effects of probiotics on food safety through anti-QS activity [42].

**Figure 3:**
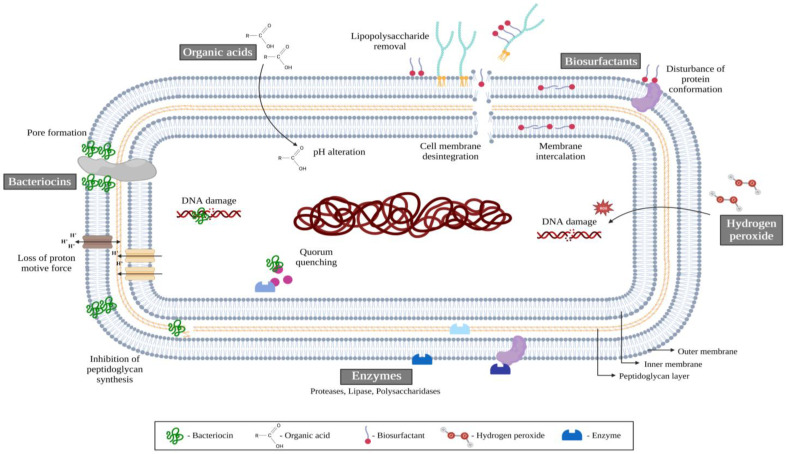
Mechanisms of action of probiotics against foodborne microorganisms. Bacteriocins can induce cell death by dissipating the proton motive force and disrupting bacterial membranes. Organic acids lead to a decrease in pH, impairing the growth of microorganisms. Biosurfactants affect cell surface compounds, causing membrane disintegration. Hydrogen peroxide can damage biomolecules such as DNA. Enzymes can target and damage extracellular proteins, exopolysaccharides, DNA, or QS molecules. Adapted from [30,43].

Figure 3 illustrates the hypothetical mechanisms of action of probiotics in controlling and preventing biofilm formation by foodborne pathogens. Bacteriocins can compromise cell integrity by dissipating the proton motive force and disrupting bacterial membranes through pore formation or inhibition of peptidoglycan synthesis. Organic acids, such as lactic acid, lead to a lowering of pH that can inhibit the growth of microorganisms without affecting the probiotics due to their tolerance to low pH [30]. Biosurfactants can affect cell surface compounds (e.g., surface proteins) and remove lipopolysaccharides from Gram-negative bacteria, decreasing the cell surface hydrophobicity and preventing further bacterial adhesion to food contact surfaces [43]. Hydrogen peroxide is an oxygen-containing compound with reactive properties that can damage biomolecules such as DNA [43]. Enzymes can target microorganisms for hydrolysis of extracellular proteins, degradation of exopolysaccharides, eDNA damage by endonucleases, and degradation of QS molecules by quorum-quenching enzymes. In addition to quorum-quenching enzymes, QS inhibition may occur by the repression of genes encoding QS signals or by interference with their receptors [42].

Once inside the human body, foodborne pathogens can boost their survival in the gut tract by forming biofilms, the formation of which is regulated by QS. Thus, probiotics (ingested, for example, in fermented foods) play a dual role in both food safety and quality and in gut health, possibly by disrupting the QS activity of pathogenic bacteria [42].

**Figure 4:**
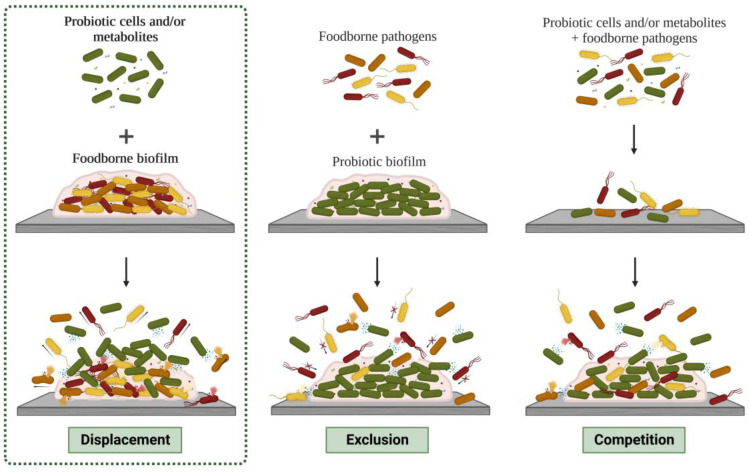
Schematic representation of the inhibition strategies of foodborne biofilms by probiotic cells and/or their metabolites: displacement, exclusion, and competition. This review addresses the displacement approach.

Probiotics can inhibit the growth of microorganisms and biofilm formation through displacement, exclusion, or competition [44], as shown in Figure 4. Displacement consists of adding probiotics and/or their metabolites to disrupt already formed biofilms; exclusion consists of coating food contact surfaces with probiotic biofilms and/or their metabolites to prevent the adhesion of pathogenic microorganisms, and competition involves the direct interaction of probiotics and/or their metabolites with foodborne microorganisms [30,45]. For a more detailed discussion of these strategies, the reader is referred to reviews on this matter [44,45].

## 5. Probiotic Displacement Effects

The use of probiotics to control and prevent biofilm formation has been increasingly researched in the food industry. In this review, we summarised the most relevant studies on the displacement strategy, grouping them according to the probiotic genus (Table 1). The most frequently tested probiotic genera were *Lactiplantibacillus* and *Lacticaseibacillus*. Among the articles summarised in Table 1, the commonly used methodologies for biofilm analysis were crystal violet (CV) staining (used in 19 of the 29 studies performing displacement anti-biofilm assays) and colony forming units (CFU) counting (performed in 10 studies). Several materials were assessed for biofilm formation, with glass and polystyrene being the most used (in 8 studies each), followed by stainless steel (used in 5 studies). Polyvinyl chloride, polytetrafluoroethylene, wood, rubber, and silicone have also been investigated. Microtiter plates were the most used biofilm formation platform (86% of the studies). Of the 29 analysed studies, 12 only reported the biofilm platform employed (microtiter plate) without specifying its material, and one included study did not report the material or the biofilm platform used. Regarding the anti-biofilm compounds evaluated, cell-free supernatant (CFS) and probiotic cells were the most tested (34 and 31% of the studies, respectively), followed by bacteriocins, crude extracts, biosurfactants, and exopolysaccharides (EPS).

The reviewed articles differed substantially in the conditions in which anti-biofilm assays were performed, which led to some discrepancies between studies that evaluated the same probiotic(s) and pathogen(s). The aforementioned experimental conditions include (1) the culture medium and the contact time between probiotics and pathogens (ranging from 5 min to 3 weeks), which may influence the growth of microorganisms and the metabolites produced, and therefore, the ability of probiotics to displace mature biofilms; (2) the duration of pathogen biofilm formation (ranging from 1.5 h to 6 days); (3) the temperature conditions (ranging from 4 °C to 42 °C, with the most common temperature being 37 °C); (4) the material tested, which may affect microorganism adhesion, since this depends on surface rigidity, coarseness, material composition, and topography [18]; (5) the initial cell concentration; and (6) the methodology used for biofilm analysis. 

### 5.1. Lactiplantibacillus *spp*.

The anti-biofilm activity of *Lactiplantibacillus* cells was evaluated against *Bacillus cereus*, *S. aureus*, *E. coli*, *Pseudomonas aeruginosa* and *Salmonella typhimurium* [46,47]. No repression of *B. cereus* was obtained. In contrast, *S. aureus* biofilms were reduced by up to 100%, and the removal rates of established biofilms of *E. coli* and *P. aeruginosa* were higher than 94% and 99.9%, respectively. Gavrilova et al. [46] reported that the production of various organic acids leading to broth acidification was responsible for the effect against pre-established biofilms of these pathogens. Indeed, the antagonistic effect of probiotic cells depends on the metabolites produced and secreted, and the competition for nutrients [18]. 

Different concentrations of bacteriocins produced by *Lactiplantibacillus plantarum* strains were able to disrupt pre-formed biofilms of *L. monocytogenes* [48,49]. Most cell membranes were damaged after treatment with bacteriocins, and intracellular constituents leaked. In addition, bacteriocins could impair the synthesis of *L. monocytogenes* proteins and cell motility [49]. Other proposed mechanisms for the anti-biofilm activity of bacteriocins are pore formation, membrane disintegration with loss of essential compounds and ions, and interference with bacterial DNA and QS system [72,73].

The metabolites produced by probiotics are secreted into the medium and can be collected in the CFS. CFS from *Lactiplantibacillus* strains induced the dispersion of *P. aeruginosa* and *L. monocytogenes* biofilms to varying degrees [51,52]. The pH of the CFS tested against *P. aeruginosa* was neutralised, thereby excluding the activity of organic acids. Thus, the anti-biofilm activity may be attributed to bacteriocin-like substances or biosurfactants [51]. Contrarily, *S. typhimurium* biofilms grew upon contact with CFS due to the inability of the anti-biofilm compounds to diffuse into the biofilm matrix [50]. 

Xinran et al. [53] evaluated the activity of a crude extract from *Lactiplantibacillus plantarum* against *Aeromonas sobria*, a pathogen commonly found in aquaculture environments. The crude extract consisted of the metabolic components of CFS extracted with ethyl acetate. Biofilm removal of up to 66% was observed, possibly due to the reduction in the levels of some virulence factors, thus suppressing QS. Besides, treatment with the crude extract resulted in a significant reduction in bacterial swimming and swarming [53].

### 5.2. Lacticaseibacillus *spp*.

Regarding *Lacticaseibacillus*, lower biofilm reduction was observed for *S. typhimurium* (up to 0.4 log) compared to *L. monocytogenes* (up to 4 log) [54]. CFS from probiotic strains of this genus showed marked anti-biofilm activity against several pathogens. The biomass and metabolic activity of biofilms of *Vibrio parahaemolyticus* were reduced by 20 and 41%, respectively [55]. Reductions of 65–77%, 58–84%, and 28–63% were obtained for mature biofilms of *S. aureus*, *E. coli*, and *Acinetobacter baumannii* [3,56]. Mobin et al. [56] stated that the anti-biofilm activity against *S. aureus* could be related to lactic acid production, with no involvement of bacteriocins. CFS also induced the biofilm disruption of *Cronobacter sakazakii* (10–51%) and *L. monocytogenes* (16–52%) [57,58]. Although the biofilm inhibitory activity was weakened after pH neutralisation of CFS, significant dispersion was still detected [57,58], as well as after CFS treatment with heat or endopeptidases [58]. This suggests the role of other antimicrobial agents in addition to organic acids, such as bacteriocins and antibacterial enzymes. CFS also contained a potent surfactant called laurostearic acid, comprising 1.8% of CFS content, which can have contributed to biofilm removal activity [58]. Biosurfactants extracted from *Lacticaseibacillus* cultures dispersed the established biofilms of *S. aureus*, *E. coli*, *Bacillus subtilis*, and *P. aeruginosa* [59,60]. These metabolites disrupt the cell membranes leading to leakage and further cell death [60]. Moreover, other mechanisms of action of biosurfactants include disturbance of protein conformation and cell division cycle [74].

### 5.3. Lactobacillus *spp*.

Similar to *Lacticaseibacillus*, *Lactobacillus* planktonic cells were less effective against *S. typhimurium* biofilms (0.8 log cell reduction) than *L. monocytogenes* (1.5 log reduction) [54]. CFS from *Lactobacillus* induced biofilm disruption, reducing the cell density of *S. aureus*, *P. aeruginosa* and *L. monocytogenes* biofilms between 18% and 87% [56,58,61]. Mobin et al. [56] reported that the antibacterial activity against *S. aureus* was not related to bacteriocin production since it has been demonstrated that the LAB strain studied only produces lactacin B when the bacteria grow in co-culture. Likewise, Niharika et al. [61] stated that the deleterious effect of CFS on sessile cells of *P. aeruginosa* and *S. aureus* was mainly due to the presence of lactic acid, as upon neutralisation, the anti-biofilm activity was lower. The remaining anti-biofilm activity may be due to the presence of sodium lactate (a neutralised form of lactic acid). CFS reduced the biofilm biomass, and microcolony formation, suggesting an increased detachment at later stages, possibly due to bacteriocin production. Different concentrations of biosurfactant isolated from *Lactobacillus* dispersed pre-formed biofilms of *S. aureus* at approximately 45–63% [59].

### 5.4. Limosilactobacillus *spp*.

*Limosilactobacillus* strains disrupted *S. aureus* biofilms by up to 100%, and almost no viable cells of *E. coli* and *P. aeruginosa* were detected in biofilms treated with these probiotics [46]. Broth acidification was the dominant antagonistic factor. In addition, *P. aeruginosa* biofilms were completely disrupted upon contact with the CFS of *Limosilactobacillus* [62]. This inhibitory effect may have resulted from the production of lactic, acetic, and formic acids or bacteriocins that are active under acidic conditions. CFS could also disperse mature biofilms of *E. coli* (58–84%) and *A. baumannii* (28–63%) [3]. Mohammed et al. [63] demonstrated that crude extract from *Limosilactobacillus* reduced *Chromobacterium violaceum* and *P. aeruginosa* by up to 40 and 32%, respectively, mainly due to EPS and other metabolites showing QS inhibitory properties.

### 5.5. Ligilactobacillus *spp*., Lactilactobacillus *spp*., Levilactobacillus *spp*. and Companilactobacillus *spp*.

Other lactobacilli have also been tested for anti-biofilm activity but against fewer pathogens. *L. monocytogenes* biofilm was depleted by 63% using *Ligilactobacillus* CFS [58]. *Lactilactobacillus* can also reduce the mature biofilm of this pathogen, while its bacteriocin extract has shown a higher anti-biofilm effect (about 3 log reduction) [64]. In contrast, a bacteriocin from *Levilactobacillus* did not significantly impair *E. coli* and *S. typhimurium* biofilms [31]. A crude extract of *Companilactobacillus* reduced the biofilm biomass and culturable cells of *P. aeruginosa* by up to 39 and 98%, respectively, as shown by Cui et al. [65]. These authors suggested that the crude extract might downregulate two pairs of QS regulatory genes and inhibit bacterial swimming and swarming.

### 5.6. Pediococcus *spp*., Leuconostoc *spp*., Lactococcus *spp*. and Enterococcus *spp*.

Other LAB, such as *Pediococcus* and *Leuconostoc*, also showed good anti-biofilm performance. *Pediococcus* cultures exhibited a significant inhibition effect against *S. typhimurium*, *L. monocytogenes*, *E. coli* and *S. aureus*, reducing pre-formed biofilms by 3–4 log CFU per coupon of polyvinyl chloride, stainless steel or glass [18]. The formation of an acidic environment may influence pathogen aggregation and attenuate biofilm formation or directly affect cell metabolism, thereby causing cell death. Additionally, *S. typhimurium* biofilm formation was reduced by 33% when treated with CFS from *Pediococcus* through extracellular matrix disruption by the bacteriocin produced [50]. Crude extracts of *Pediococcus* dispersed *C. violaceum* and *P. aeruginosa* biofilms by up to 40 and 32%, respectively [63].

EPS isolated from *Pediococcus* and *Leuconostoc* showed antimicrobial activity against Gram-positive and Gram-negative bacteria [66]. Their capacity to disperse mature biofilms was demonstrated against *S. aureus*, *E. coli*, and *Enterococcus faecalis* (33–80% biomass reduction). The disruptive effect increased when increasing EPS concentrations were added. It has been reported that EPS from LAB can affect bacterial surface properties by weakening cell-surface modifications and cell-cell interactions. Through these antagonistic properties, EPS appear to have a greater ability to inhibit the initial attachment and autoaggregation (in competition and exclusion strategies) compared to the disruption of mature biofilms [66].

LAB from *Lactococcus* and *Enterococcus* genera substantially decreased the number of culturable biofilm cells of *L. monocytogenes* in multispecies biofilms (up to 6 and 5 log reduction, respectively) upon treatment at 4 °C or 8 °C, the temperature at which probiotic growth would be slowed or suppressed [67]. Another study revealed that *L. monocytogenes* biofilms decreased by 2.7 log when exposed to a bacteriocin solution from *Lactococcus* [68].

### 5.7. Bifidobacterium *spp*. and Saccharomyces *spp*.

The supernatants from *Bifidobacterium* did not impair mature *S. typhimurium* biofilms because the antimicrobial compounds produced were unable to diffuse into the biofilm structure [50]. In contrast, CFS from *Saccharomyces* spp. dispersed approximately 52–77% of the cells attached to glass coupons, depending on the CFS concentration used [69].

### 5.8. Probiotic Cocktails

Some authors have previously tested probiotic cocktails. Moradi et al. [70] reported that a cocktail of *Lactobacillus animalis*, *Lactobacillus amylovorus*, and *Pediococcus acidilacti* cells significantly reduced *L. monocytogenes* biofilm culturability after 24 h of contact (98%), and was still able to displace *L. monocytogenes* cells attached to the surface after 72 h. The inhibition of pathogen cells from accessing available nutrients could be the factor behind the remarkable antagonistic activity observed. In turn, Monteiro et al. [71] studied the anti-biofilm activity of a mixture of *Bacillus* and *Pediococcus* species against *Salmonella heidelberg*, *Salmonella gallinarum*, *S. aureus*, and *Campylobacter jejuni*. The mature biofilms were reduced by up to 99.99% for *S. heidelberg*, *S. gallinarum* and *S. aureus* and by up to 14.9% for *C. jejuni*, depending on the material used (soil, wood, and polystyrene) and the contact time.

## 6. Conclusions

The emergence of resistant pathogenic bacteria throughout the food chain emphasises the need to explore alternatives for disinfection. Therefore, there is great interest in the development of novel strategies using natural products to control the persistence of pathogens associated with food surfaces or equipment without conferring an added risk to consumers. Probiotics and/or their secreted compounds have shown great potential to disrupt pre-formed biofilms of a large spectrum of foodborne microorganisms. *L. monocytogenes* and *S. aureus* are the most studied biofilm-forming pathogens, while *Lactiplantibacillus* and *Lacticaseibacillus* are the most tested probiotic genera. Probiotic cells and cell-free supernatants are the most used agents to displace biofilms from abiotic surfaces. Their mechanisms of action depend on secreted metabolites (e.g., bacteriocins, organic acids, and biosurfactants) and competition for nutrients in the case of whole cells. Whole cells revealed the most promising results in biofilm displacement among the antimicrobial substances tested, followed by bacteriocins. However, as anti-biofilm assays were performed under non-standardised conditions, it is difficult to compare the efficacy of the same probiotic(s) and pathogen(s). It is believed that the standardisation of anti-biofilm tests for evaluating the potential of probiotics to control biofilm development in food industries is of extreme importance to obtain more reliable, comparable, and predictable results.

Although the reviewed studies show encouraging results, to the best of our knowledge, only two were performed in real food processing facilities, indicating that the use of probiotics for biofilm control may be far from a practical application. More in situ studies employing the active microflora present in each food environment are necessary to better evaluate the efficacy of probiotics and/or their metabolites to control unwanted biofilm formation and strengthen the in vitro outcomes. Moreover, the possibility of resistance development and adaptive evolutionary responses upon contact between probiotics and foodborne pathogens was poorly addressed, suggesting that further studies are needed to clarify these questions before applying non-pathogenic microorganisms such as probiotics as part of daily cleaning products in food processing facilities. Even though several LAB species have GRAS status (e.g., lactobacilli and lactococci), the uncontrolled release of some of the others (e.g., enterococci) may pose some public health risks, as horizontal gene transfer responsible for drug resistance and/or infectivity, excessive immune system stimulation, cytotoxicity against human cells, and gastrointestinal disturbances. 

It is believed that the safe and successful application of probiotics in food production, either alone or in combination with routine sanitisation procedures (employing chemicals and/or other agents), can reduce the risk of cross-contamination with pathogenic bacteria, thus limiting foodborne outbreaks and improving public health in a sustainable and environmentally friendly way.

## Figures and Tables

**Table 1:** Ability of probiotics and/or their metabolites to control biofilms formed in food context through the displacement strategy.

Probiotic Genus	Anti-Biofilm Compound	Foodborne Microorganism	Outcomes	References
*Lactiplantibacillus*	Cells	*Bacillus cereus*	No repression of *B. cereus* was observed. In contrast, treated *S. aureus* biofilms were reduced by up to 100%. Likewise, the removal rates of established biofilms of *E. coli* and *P. aeruginosa* were higher than 93.7% and 99.9%, respectively. A remarkable biofilm reduction of 99.6% was observed for *S. typhimurium*.	[46,47]
		*Staphylococcus aureus*		
		*Escherichia coli*		
		*Pseudomonas aeruginosa*		
		*Salmonella typhimurium*		
	Bacteriocin	*Listeria monocytogenes*	Treatment with bacteriocins completely disintegrated *L. monocytogenes* biofilms and caused extensive cell membrane damage. High concentrations of bacteriocins were more effective in removing biofilms.	[48,49]
	CFS	*S. typhimurium*	CFS promoted biofilm growth.	[50]
		*P. aeruginosa*	*P. aeruginosa* biofilms were reduced by 15–78% in a concentration-dependent manner.	[51]
		*L. monocytogenes*	Biofilm reductions of up to 90% were obtained upon treatment with CFS.	[52]
	Crude extract	*Aeromonas sobria*	Biofilm removal of up to 66% was observed, possibly due to the reduction in the levels of some virulence factors, thus suppressing QS.	[53]
*Lacticaseibacillus*	Cells	*S. typhimurium*	The displacement activity of the probiotic planktonic cells was not effective against *S. typhimurium* (up to 0.4 log reduction). Biofilm dispersion was more pronounced for *L. monocytogenes* (99.9%).	[54]
		*L. monocytogenes*		
	CFS	*Vibrio parahaemolyticus*	Biofilm biomass and metabolic activity were reduced by up to 41%.	[55]
		*S. aureus*	CFS significantly removed the pre-established biofilm in the range of 65–77%.	[56]
		*E. coli*	Significant eradication of established biofilms of both *E. coli* (58–84%) and *A. baumannii* (28–63%) was observed after treatment with the CFS.	[3]
		*Acinetobacter baumannii*		
		*Cronobacter sakazakii*	The reduction of pre-formed biofilms was around 10–51% for *C. sakazakii* and 16–52% for *L. monocytogenes,* depending on the contact time and CFS concentration.	[57,58]
		*L. monocytogenes*		
	Biosurfactant	*S. aureus*	Biosurfactants extracted from *Lacticaseibacillus* dispersed the pre-formed biofilms in a dose-dependent manner, ranging from 48% to 76%.	[59,60]
		*E. coli*		
		*Bacillus subtilis*		
		*P. aeruginosa*		
*Lactobacillus*	Cells	*S. typhimurium*	The displacement activity of probiotic cells was not effective against *S. typhimurium* (0.8 log reduction). Biofilm dispersion was more pronounced against *L. monocytogenes* (97%).	[54]
		*L. monocytogenes*		
	CFS	*S. aureus*	CFS reduced the population of sessile cells of *P. aeruginosa* by up to 77% and removed *S. aureus* biofilms by approximately 18–87%. Following neutralisation, a marked reduction in biofilm inhibition was observed.	[56,61]
		*P. aeruginosa*		
		*L. monocytogenes*	CFS reduced the biofilm amount of *L. monocytogenes* by 48%.	[58]
	Biosurfactant	*S. aureus*	Biosurfactants extracted from the probiotic strains dispersed the pre-formed biofilms around 45–63%.	[59]
*Limosilactobacillus*	Cells	*B. cereus*	Whereas *B. cereus* biofilms were not affected, viable *S. aureus* cells were not detected in the biofilms exposed to the probiotics. The removal values of the pre-established *E. coli* and *P. aeruginosa* biofilms were higher than 6 and 5 log CFU/mL, respectively.	[46]
		*S. aureus*		
		*E. coli*		
		*P. aeruginosa*		
	CFS	*P. aeruginosa*	The CFS of *Limosilactobacillus* totally removed the pre-formed biofilms of *P. aeruginosa.*	[62]
		*E. coli*	Significant eradication of established biofilms of both *E. coli* (58–84%) and *A. baumannii* (28–63%) was observed after treatment with CFS.	[3]
		*A. baumannii*		
	Crude extract	*Chromobacterium violaceum*	The crude extract exhibited significant QS inhibitory and anti-biofilm properties, reducing *C. violaceum* biofilm by 3 to 40% and *P. aeruginosa* biofilm by up to 32%.	[63]
		*P. aeruginosa*		
*Ligilactobacillus*	CFS	*L. monocytogenes*	*L. monocytogenes* biofilm was depleted by 63% with *Ligilactobacillus* CFS.	[58]
*Lactilactobacillus*	Cells	*L. monocytogenes*	*Lactilactobacillus* cells were able to displace the pre-established biofilm by 1.8–2.2 log CFU/cm^2^. An enhanced pathogen inhibition was observed when the semi-purified bacteriocin extract was added (reductions of 3.1–3.6 log CFU/cm^2^).	[64]
	Bacteriocin			
	CFS	*S. typhimurium*	CFS increased the *S. typhimurium* biofilm due to the inability of the anti-biofilm substances to diffuse through the biofilm matrix.	[50]
*Levilactobacillus*	Bacteriocin	*E. coli*	Bacteriocins did not significantly remove the biofilms of *E. coli* or *S. typhimurium* (only 16%).	[31]
		*S. typhimurium*		
	CFS	*E. coli*	Significant eradication of established biofilms of both *E. coli* (58–84%) and *A. baumannii* (28–63%) was observed.	[3]
		*A. baumannii*		
*Companilactoba* *cillus*	Crude extract	*P. aeruginosa*	Crude extract of *Companilactobacillus* had a strong removal effect on *P. aeruginosa* biofilms, achieving up to 39% biomass removal and 98% CFU reduction.	[65]
*Pediococcus*	Cells	*S. aureus*	*Pediococcus* had a significant inhibition effect in the displacement strategy, with a reduction of 3–4 log CFU/coupon.	[18]
		*S. typhimurium*		
		*L. monocytogenes*		
		*E. coli*		
	CFS	*S. typhimurium*	*Pediococcus* caused a statistically significant removal (33%) of mature *S. typhimurium* biofilms.	[50]
	Crude extract	*C. violaceum*	The crude extract exhibited significant QS inhibitory and anti-biofilm properties, reducing *C. violaceum* and *P. aeruginosa* biofilms by up to 40% and 32%, respectively.	[63]
		*P. aeruginosa*		
	EPS	*S. aureus*	*S. aureus*, *E. coli*, and *E. faecalis* mature biofilms were reduced by up to 75%, 52% and 50%, respectively, and the disruptive activity increased with increasing EPS concentration.	[66]
		*E. coli*		
		*Enterococcus faecalis*		
*Lactococcus*	Cells	*L. monocytogenes*	*L. monocytogenes* biofilms decreased by 1–6 log and 2.7 log when exposed to cells and bacteriocin from *Lactococcus*, respectively.	[67,68]
	Bacteriocin			
*Leuconostoc*	EPS	*S. aureus*	*S. aureus*, *E. coli,* and *E. faecalis* biofilms were reduced by up to 77%, 62% and 53%, respectively, and the disruptive activity increased with increasing EPS concentration.	[66]
		*E. coli*		
		*E. faecalis*		
*Enterococcus*	Cells	*L. monocytogenes*	*L. monocytogenes* biofilms decreased by 1–5 log.	[67]
*Bifidobacterium*	CFS	*S. typhimurium*	The CFS of *Bifidobacterium* did not affect mature *S. typhimurium* biofilms.	[50]
*Saccharomyces*	CFS	*L. monocytogenes*	The structure of CFS-treated *L. monocytogenes* biofilms was dispersed, and the number of cells attached to the surface decreased by 52–77%.	[69]
Cocktail (*Lactobacillus* and *Pediococcus*)	Cells	*L. monocytogenes*	The LAB cocktail significantly displaced *L. monocytogenes* biofilms after a 24 h contact time (98%) and was still able to reduce mature biofilms after 72 h.	[70]
Cocktail (*Bacillus* and *Pediococcus*)	Cells	*Salmonella gallinarum*	Biofilm reductions of up to 99.9% were detected.	[71]
		*Salmonella heidelberg*		
		*S. aureus*		
		*Campylobacter jejuni*		

Abbreviations: CFS, Cell-Free Supernatant; CFU, Colony Forming Units; EPS, Exopolysaccharides; LAB, Lactic Acid Bacteria; QS, Quorum-Sensing.

## Data Availability

Not applicable.

## References

[1] Tazehabadi M.H., Algburi A., Popov I.V., Ermakov A.M., Chistyakov V.A., Prazdnova E.V., Weeks R., Chikindas M.L. (2021). Probiotic Bacilli Inhibit *Salmonella* Biofilm Formation Without Killing Planktonic Cells. Front. Microbiol..

[2] Gutiérrez S., Martínez-Blanco H., Rodríguez-Aparicio L.B., Ferrero M.A. (2016). Effect of fermented broth from lactic acid bacteria on pathogenic bacteria proliferation. J. Dairy Sci..

[3] Sornsenee P., Chatatikun M., Mitsuwan W., Kongpol K., Kooltheat N., Sohbenalee S., Pruksaphanrat S., Mudpan A., Romyasamit C. (2021). Lyophilized cell-free supernatants of *Lactobacillus* isolates exhibited antibiofilm, antioxidant, and reduces nitric oxide activity in lipopolysaccharide-stimulated RAW 264.7 cells. PeerJ.

[4] Bermudez-Brito M., Plaza-Díaz J., Muñoz-Quezada S., Gómez-Llorente C., Gil A. (2012). Probiotic Mechanisms of Action. Ann. Nutr. Metab..

[5] Divyashree S., Anjali P.G., Somashekaraiah R., Sreenivasa M.Y. (2021). Probiotic properties of *Lactobacillus casei*—MYSRD 108 and *Lactobacillus plantarum*—MYSRD 71 with potential antimicrobial activity against *Salmonella paratyphi*. Biotechnol. Rep..

[6] Qualified Presumption of Safety (QPS): EFSA Journal. https://efsa.onlinelibrary.wiley.com/doi/toc/10.1002/(ISSN)1831-4732.QPS.

[7] EUR-Lex—Regulation (EC) No 1924/2006 of the European Parliament and of the Council of 20 December 2006 on Nutrition and Health Claims Made on Foods. https://eur-lex.europa.eu/legal-content/en/ALL/?uri=CELEX%3A32006R1924.

[8] Global Overview for Probiotics: Trends, Markets, and Harmonization|RAPS. https://www.raps.org/news-and-articles/news-articles/2022/9/global-overview-for-probiotics-trends-markets-and?GA_network=x&GA_device=c&GA_campaign=18448087812&GA_adgroup=&GA_target=&GA_placement=&GA_creative=&GA_extension=&GA_keyword=&GA_loc_physical_ms=1011759&GA_landingpage=https\%3a\%2fwww.raps.org\%2fnews-and-articles\%2fnews-articles\%2f2022\%2f9\%2fglobal-overview-for-probiotics-trends-markets-and&gclid=Cj0KCQiAiJSeBhCCARIsAHnAzT-P6bWY8j7jWWPwV1asExzYsInprUJ6LfBJ3im7LfLWkMb7cPA_OQAaAm_UEALw_wcB.

[9] Moradi M., Kousheh S.A., Almasi H., Alizadeh A., Guimarães J.T., Yılmaz N., Lotfi A. (2020). Postbiotics produced by lactic acid bacteria: The next frontier in food safety. Compr. Rev. Food Sci..

[10] Asaithambi N., Singh S.K., Singha P. (2021). Current status of non-thermal processing of probiotic foods: A review. J. Food Eng..

[11] Global and European Probiotic Market Insights 2018–2021. https://www.ipaeurope.org/wp-content/uploads/2022/05/Market-data-probiotics-2018-2021.pdf.

[12] The Burden of Foodborne Diseases in the WHO European Region. https://www.euro.who.int/__data/assets/pdf_file/0005/402989/50607-WHO-Food-Safety-publicationV4_Web.pdf.

[13] Hossain M.I., Mizan M.F.R., Roy P.K., Nahar S., Toushik S.H., Ashrafudoulla M., Jahid I.K., Lee J., Ha S.-D. (2021). *Listeria monocytogenes* biofilm inhibition on food contact surfaces by application of postbiotics from *Lactobacillus curvatus* B.67 and *Lactobacillus plantarum* M.2. Food Res. Int..

[14] Qiao Z., Chen J., Zhou Q., Wang X., Shan Y., Yi Y., Liu B., Zhou Y., Lü X. (2021). Purification, characterization, and mode of action of a novel bacteriocin BM173 from *Lactobacillus crustorum* MN047 and its effect on biofilm formation of *Escherichia coli* and *Staphylococcus aureus*. J. Dairy Sci..

[15] Jaffee S., Henson S., Unnevehr L., Grace D., Cassou E. (2019). The Safe Food Imperative: Accelerating Progress in Low- and Middle-Income Countries.

[16] USDA ERS—Cost Estimates of Foodborne Illnesses. https://www.ers.usda.gov/data-products/cost-estimates-of-foodborne-illnesses.aspx.

[17] The European Union One Health 2020 Zoonoses Report. https://efsa.onlinelibrary.wiley.com/doi/full/10.2903/j.efsa.2021.6971.

[18] Tan X., Han Y., Xiao H., Zhou Z. (2017). *Pediococcus acidilactici* Inhibit Biofilm Formation of Food-Borne Pathogens on Abiotic Surfaces. Trans. Tianjin Univ..

[19] Sahoo M., Panigrahi C., Aradwad P. (2022). Management strategies emphasizing advanced food processing approaches to mitigate food borne zoonotic pathogens in food system. Food Front..

[20] EUR-Lex—Council Regulation (EEC) No 315/93 of 8 February 1993 Laying down Community Procedures for Contaminants in Food. https://eur-lex.europa.eu/legal-content/EN/ALL/?uri=celex%3A31993R0315.

[21] Hadawey A., Savvas A.T., Chaer I., Sundararajan R. (2017). Unwrapped food product display shelf life assessment. Energy Procedia.

[22] Coughlan L.M., Cotter P.D., Hill C., Alvarez-Ordóñez A. (2016). New Weapons to Fight Old Enemies: Novel Strategies for the (Bio)control of Bacterial Biofilms in the Food Industry. Front. Microbiol..

[23] Carrascosa C., Raheem D., Ramos F., Saraiva A., Raposo A. (2021). Microbial Biofilms in the Food Industry—A Comprehensive Review. Int. J. Environ. Res. Public Health.

[24] Jara J., Pérez-Ramos A., del Soar G., Rodríguez J.M., Fernández L., Orgaz B. (2020). Role of *Lactobacillus* biofilms in *Listeria monocytogenes* adhesion to glass surfaces. Int. J. Food Microbiol..

[25] Galié S., García-Gutiérrez C., Miguélez E.M., Villar C.J., Lombó F. (2018). Biofilms in the Food Industry: Health Aspects and Control Methods. Front. Microbiol..

[26] Dhivya R., Rajakrishnapriya V.C., Sruthi K., Chidanand D.V., Sunil C.K., Rawson A. (2022). Biofilm combating in the food industry: Overview, non-thermal approaches, and mechanisms. J. Food Process. Preserv..

[27] Azeredo J., Azevedo N.F., Briandet R., Cerca N., Coenye T., Costa A.R., Desvaux M., Bonaventura G.D., Hébraud M., Jaglic Z. (2016). Critical review on biofilm methods. Crit. Rev. Microbiol..

[28] Stoodley P., Sauer K., Davies D.G., Costerton J.W. (2002). Biofilms as Complex Differentiated Communities. Annu. Rev. Microbiol..

[29] Petrova O.E., Sauer K. (2012). Sticky Situations: Key Components That Control Bacterial Surface Attachment. J. Bacteriol..

[30] Toushik S.H., Kim K.-S., Ashrafudoulla M., Mizan M.F.R., Roy P.K., Nahar S., Kim Y., Ha S.D. (2021). Korean kimchi-derived lactic acid bacteria inhibit foodborne pathogenic biofilm growth on seafood and food processing surface materials. Food Control.

[31] Kim N.-N., Kim W.J., Kang S.-S. (2019). Anti-biofilm effect of crude bacteriocin derived from *Lactobacillus brevis* DF01 on *Escherichia coli* and *Salmonella Typhimurium*. Food Control.

[32] Cisneros L., Cattelan N., Villalba M.I., Rodriguez C., Serra D.O., Yantorno O., Fadda S. (2021). Lactic acid bacteria biofilms and their ability to mitigate *Escherichia coli* O157:H7 surface colonization. Lett. Appl. Microbiol..

[33] Zhu T., Yang C., Bao X., Chen F., Guo X. (2022). Strategies for controlling biofilm formation in food industry. Grain Oil Sci. Technol..

[34] Flemming H.-C., Wingender J., Szewzyk U., Steinberg P., Rice S.A., Kjelleberg S. (2016). Biofilms: An emergent form of bacterial life. Nat. Rev. Microbiol..

[35] Sauer K., Stoodley P., Goeres D.M., Hall-Stoodley L., Burmlle M., Stewart P.S., Bjarnsholt T. (2022). The biofilm life cycle: Expanding the conceptual model of biofilm formation. Nat. Rev. Microbiol..

[36] Merino L., Procura F., Trejo F.M., Bueno D.J., Golowczyc M.A. (2019). Biofilm formation by *Salmonella* sp. in the poultry industry: Detection, control and eradication strategies. Food Res. Int..

[37] Wang N., Yuan L., Sadiq F.A., He G. (2019). Inhibitory effect of *Lactobacillus plantarum* metabolites against biofilm formation by *Bacillus licheniformis* isolated from milk powder products. Food Control.

[38] Kıran F., Akoğlu A., Çakır İ. (2021). Control of *Listeria monocytogenes* biofilm on industrial surfaces by cell-free extracts of *Lactobacillus plantarum*. J. Food Process. Preserv..

[39] El-Tarabily K.A., El-Saadony M.T., Alagawany M., Arif M., Batiha G.E., Khafaga A.F., Elwan H.A.M., Elnesr S.S., Abd El-Hack E.M. (2021). Using essential oils to overcome bacterial biofilm formation and their antimicrobial resistance. Saudi J. Biol. Sci..

[40] Dobson A., Cotter P.D., Ross R.P., Hill C. (2012). Bacteriocin Production: A Probiotic Trait?. Appl. Environ. Microbiol..

[41] Soltani S., Biron E., Said L.B., Subirade M., Fliss I. (2022). Bacteriocin-Based Synergetic Consortia: A Promising Strategy to Enhance Antimicrobial Activity and Broaden the Spectrum of Inhibition. Microbiol. Spectr..

[42] Salman M.K., Abuqwider J., Mauriello G. (2023). Anti-Quorum Sensing Activity of Probiotics: The Mechanism and Role in Food and Gut Health. Microorganisms.

[43] Toushik S.H., Mizan M.F.R., Hossain M.I., Ha S.-D. (2020). Fighting with old foes: The pledge of microbe-derived biological agents to defeat mono- and mixed-bacterial biofilms concerning food industries. Trends Food Sci. Technol..

[44] Carvalho F.M., Teixeira-Santos R., Mergulhão F.J.M., Gomes L.C. (2021). Targeting biofilms in medical devices using probiotic cells: A systematic review. AIMS Mater. Sci..

[45] Carvalho F.M., Teixeira-Santos R., Mergulhão F.J.M., Gomes L.C. (2021). The Use of Probiotics to Fight Biofilms in Medical Devices: A Systematic Review and Meta-Analysis. Microorganisms.

[46] Gavrilova E., Anisimova E., Gabdelkhadieva A., Nikitina E., Vafina A., Yarullina D., Bogachev M., Kayumov A. (2019). Newly isolated lactic acid bacteria from silage targeting biofilms of foodborne pathogens during milk fermentation. BMC Microbiol..

[47] Ruiz M.J., García M.D., Padola N.L., Etcheverría A.I. (2022). Ability of *Lactiplantibacillus plantarum* to reduce biofilms of pathogens involved in foodborne diseases. Rev. Vet..

[48] Todorov S.D., de Paula O.A.L., Camargo A.C., Lopes D.A., Nero L.A. (2018). Combined effect of bacteriocin produced by *Lactobacillus plantarum* ST8SH and vancomycin, propolis or EDTA for controlling biofilm development by *Listeria monocytogenes*. Rev. Argent. Microbiol..

[49] Liu Y., Bu Y., Li J., Liu Y., Liu A., Gong P., Liu T., Zhang L., Wang S., Yi H. (2022). Inhibition Activity of Plantaricin Q7 Produced by *Lactobacillus plantarum* Q7 against *Listeria monocytogenes* and Its Biofilm. Fermentation.

[50] Göksel Ş., Akçelik N., Özdemir C., Akçelik M. (2022). The Effects of Lactic Acid Bacteria on *Salmonella* Biofilms. J. Microbiol..

[51] Rao K.P., Kumar N.H., Somashekaraiah R., Murali M., Sreenivasa M.Y. (2021). Probiotic Attributes and Inhibitory Effects of *Lactobacillus plantarum* MYS84 against the Growth and Biofilm Formation of *Pseudomonas aeruginosa*. Microbiology.

[52] Ben Slama R., Kouidhi B., Zmantar T., Chaieb K., Bakhrouf A. (2013). Anti-listerial and Anti-biofilm Activities of Potential Probiotic *Lactobacillus* Strains Isolated from Tunisian Traditional Fermented Food. J. Food Saf..

[53] Lv X., Cui T., Du H., Sun M., Bai F., Li J., Zhang D. (2021). *Lactobacillus plantarum* CY 1-1: A novel quorum quenching bacteria and anti-biofilm agent against *Aeromonas sobria*. LWT.

[54] Woo J., Ahn J. (2013). Probiotic-mediated competition, exclusion and displacement in biofilm formation by food-borne pathogens. Lett. Appl. Microbiol..

[55] Shangguan W., Xie T., Zhang R., Lu C., Han X., Zhong Q. (2021). Anti-biofilm potential of kefir-derived *Lactobacillus paracasei* L10 against *Vibrio parahaemolyticus*. Lett. Appl. Microbiol..

[56] Koohestani M., Moradi M., Tajik H., Badali A. (2018). Effects of cell-free supernatant of *Lactobacillus acidophilus* LA5 and *Lactobacillus casei* 431 against planktonic form and biofilm of *Staphylococcus aureus*. Vet. Res. Forum.

[57] Singh N., Kaur R., Singh B.P., Rokana N., Goel G., Puniya A.K., Panwar H. (2020). Impairment of *Cronobacter sakazakii* and *Listeria monocytogenes* biofilms by cell-free preparations of lactobacilli of goat milk origin. Folia Microbiol..

[58] Moradi M., Mardani K., Tajik H. (2019). Characterization and application of postbiotics of *Lactobacillus* spp. on *Listeria monocytogenes* in vitro and in food models. LWT.

[59] Nataraj B.H., Ramesh C., Mallappa R.H. (2021). Characterization of biosurfactants derived from probiotic lactic acid bacteria against methicillin-resistant and sensitive *Staphylococcus aureus* isolates. LWT.

[60] Patel M., Siddiqui A.J., Hamadou W.S., Surti M., Awadelkareem A.M., Ashraf S.A., Alreshidi M., Snoussi M., Rizvi S.M.D., Bardakci F. (2021). Inhibition of Bacterial Adhesion and Antibiofilm Activities of a Glycolipid Biosurfactant from *Lactobacillus rhamnosus* with Its Physicochemical and Functional Properties. Antibiotics.

[61] Singh N., Sharma C., Gulhane R.D., Rokana N., Singh B.P., Puniya A.K., Attri S., Goel G., Panwar H. (2018). Inhibitory effects of lactobacilli of goat’s milk origin against growth and biofilm formation by pathogens: An in vitro study. Food Biosci..

[62] Shokri D., Khorasgani M.R., Mohkam M., Fatemi S.M., Ghasemi Y., Taheri-Kafrani A. (2018). The Inhibition Effect of Lactobacilli Against Growth and Biofilm Formation of *Pseudomonas aeruginosa*. Probiotics Antimicrob. Proteins.

[63] Aman M., Aneeqha N., Bristi K., Deeksha J., Afza N., Sindhuja V., Shastry R.P. (2021). Lactic acid bacteria inhibits quorum sensing and biofilm formation of *Pseudomonas aeruginosa* strain JUPG01 isolated from rancid butter. Biocatal. Agric. Biotechnol..

[64] Pérez-Ibarreche M., Castellano P., Leclercq A., Vignolo G. (2016). Control of *Listeria monocytogenes* biofilms on industrial surfaces by the bacteriocin-producing *Lactobacillus sakei* CRL1862. FEMS Microbiol. Lett..

[65] Cui T., Bai F., Sun M., Lv X., Li X., Zhang D., Du H. (2020). *Lactobacillus crustorum* ZHG 2-1 as novel quorum-quenching bacteria reducing virulence factors and biofilms formation of *Pseudomonas aeruginosa*. LWT.

[66] Abid Y., Casillo A., Gharsallah H., Joulak I., Lanzetta R., Corsaro M.M., Attia H., Azabou S. (2018). Production and structural characterization of exopolysaccharides from newly isolated probiotic lactic acid bacteria. Int. J. Biol. Macromol..

[67] Zhao T., Podtburg T.C., Zhao P., Chen D., Baker D.A., Cords B., Doyle M.P. (2013). Reduction by Competitive Bacteria of *Listeria monocytogenes* in Biofilms and Listeria Bacteria in Floor Drains in a Ready-to-Eat Poultry Processing Plant. J. Food Prot..

[68] García-Almendárez B.E., Cann I.K.O., Martin S.E., Guerrero-Legarreta I., Regalado C. (2008). Effect of *Lactococcus lactis* UQ2 and its bacteriocin on *Listeria monocytogenes* biofilms. Food Control.

[69] Kim Y.J., Yu H.H., Song Y.J., Park Y.J., Lee N.-K., Paik H.-D. (2021). Anti-biofilm effect of the cell-free supernatant of probiotic *Saccharomyces cerevisiae* against *Listeria monocytogenes*. Food Control.

[70] Ndahetuye J.B., Koo O.K., O’Bryan C.A., Ricke S.C., Crandall P.G. (2012). Role of Lactic Acid Bacteria as a Biosanitizer To Prevent Attachment of *Listeria monocytogenes* F6900 on Deli Slicer Contact Surfaces. J. Food Prot..

[71] Monteiro G.P., Rossi D.A., Valadares E.C., Peres P.A.B.M., Braz R.F., Notário F.O., Gomes M.M., Silva R.R., Carrijo K.F., Fonseca B.B. (2021). Lactic Bacterium and *Bacillus* Sp. Biofilms Can Decrease the Viability of *Salmonella gallinarum*, *Salmonella heidelberg*, *Campylobacter jejuni* and Methicillin Resistant *Staphylococcus aureus* on Different Substrates. Braz. J. Poult. Sci..

[72] Hossain M.I., Mizan M.F.R., Ashrafudoulla M., Nahar S., Joo H.-J., Jahid I.K., Park S.H., Kim K.-S., Ha S.-D. (2020). Inhibitory effects of probiotic potential lactic acid bacteria isolated from kimchi against *Listeria monocytogenes* biofilm on lettuce, stainless-steel surfaces, and MBEC™ biofilm device. LWT.

[73] Wei Y., Wang J., Liu Z., Pei J., Brennan C., Abd El-Aty A.M. (2022). Isolation and Characterization of Bacteriocin-Producing *Lacticaseibacillus rhamnosus* XN2 from Yak Yoghurt and Its Bacteriocin. Molecules.

[74] Carvalho F.M., Teixeira-Santos R., Mergulhão F.J.M., Gomes L.C. (2021). Effect of *Lactobacillus plantarum* Biofilms on the Adhesion of *Escherichia coli* to Urinary Tract Devices. Antibiotics.

